# Sepsis downregulates aortic Notch signaling to produce vascular hyporeactivity in mice

**DOI:** 10.1038/s41598-022-06949-3

**Published:** 2022-02-21

**Authors:** Vandana Singh, Raut Akash, Gaurav Chaudhary, Rajneesh Singh, Soumen Choudhury, Amit Shukla, Shyama N. Prabhu, Neeraj Gangwar, Satish K. Garg

**Affiliations:** 1 Smooth Muscle Pharmacology and Molecular Pharmacology Laboratory, Department of Veterinary Pharmacology and Toxicology, College of Veterinary Science and Animal Husbandry, U.P. Pandit Deen Dayal Upadhyaya Pashu Chikitsa Vigyan Vishwavidyalaya Evam Go-Anusandhan Sansthan (DUVASU), Mathura, 281001 India; 2 Department of Veterinary Pathology, College of Veterinary Science and Animal Husbandry, U.P. Pandit Deen Dayal Upadhyaya Pashu Chikitsa Vigyan Vishwavidyalaya Evam Go-Anusandhan Sansthan, Mathura, 281001 India

**Keywords:** Diseases, Pathogenesis

## Abstract

Inhibition of Notch signaling in macrophages is known to reduce inflammation, however, its role in regulating vascular hyporeactivity in sepsis is unknown. Thus we aimed to evaluate the effect of sepsis on vascular Notch signaling. Polymicrobial sepsis was induced by caecal ligation and puncture (CLP) in mice. mRNA expressions of Notch receptors (Notch1,3) and ligands (Jag1, Dll4), and downstream effector genes (Hey1, MLCK, MYPT1) were assessed by RT-qPCR. Protein level of activated Notch (NICD) was assessed by Western blot and immuno-histochemistry. Isometric tension in isolated aortic rings was measured by wire myography.CLP down-regulated aortic expression of Notch3, Jag1 and Dll4 as compared to control mice. Additionally, the protein level of NICD was found to be lesser in aortic tissue sections from CLP mice. Expression of Hey1 and MLCK were attenuated whereas MYPT1 expression was increased in septic mouse aorta. DAPT pretreatment did not improve CLP-induced vascular hyporeactivity to NA, CaCl_2_ and high K^+^ (80 mM), rather significantly attenuated the aortic response to these vasoconstrictors in control mice. Treatment with 1400 W reversed attenuated Notch3 (but not Jag1 and MLCK) expression in septic mouse aorta. In conclusion, sepsis significantly attenuated the Notch (especially Notch3) signaling in mouse aorta along with reduction in contractile gene expression and vasoconstriction response. Further, iNOS/NO pathway was involved in sepsis-induced down-regulation of Notch3 receptor. Thus systemic inhibition of Notch signaling during sepsis may have serious impact on sepsis-induced vascular hyporeactivity.

## Introduction

Sepsis, a systemic inflammatory response syndrome against microbial infection, is a leading cause of deaths in intensive care units^1^. Despite the progress in clinical and basic research, prognosis in septic patients still remains extremely poor and mortality rate varies from 15 to 40%^2,3^ and around 18 million cases of sepsis per year have been reported globally^4^. Dysregulated immune responses to infection in an immuno-compromised state coupled with vascular dysfunctions are the predominant cause of death in sepsis^5,6^. Circulatory failure in sepsis is characterized by refractory hypotension and vascular hyporeactivity to clinically used vasoconstrictors like nor-adrenaline leading to multi-organ dysfunction. Overproduction of inducible nitric oxide synthase (iNOS)-derived nitric oxide (NO) along with down-regulation and desensitization of α_1D_-adrenoceptor expression are considered to be the underlying mechanism of vascular hyporeactivity in sepsis^7,8^.

The canonical Notch signalling is an evolutionary conserved pathway that governs cell fate, proliferation, differentiation, apoptosis, angiogenesis and vascular remodelling^9^. In this cell to cell communication system, a membrane-tethered Notch ligand on one cell interacts with transmembrane Notch receptor on a juxtaposed cell. Four different types of Notch receptors (Notch1-4) and five ligands (Jag1, 2 and Dll1, 3, 4) are present in mammalian cells^10,11^. Upon ligand-receptor interaction, Notch receptor undergoes several proteolytic processes leading to release of Notch intracellular domain (NICD) to cytosol by the action of γ-secretase enzyme. The NICD then translocates into the nucleus and acts as a transcriptional regulator associated with transcriptional factor, recombination signal binding protein for immunoglobulin-κ J region (RBPJ-κ). The resulting NICD-RBPJ-κ complex subsequently activates transcription of target genes like Hes (hairy and enhancer of split) and Hey (Hes-related transcriptional factor)^11,12^.

Besides its major role in embryonic development, Notch pathway is also involved in many patho-physiological processes even in adult life. Pharmacological interventions in Notch signalling in different disease conditions and clinical trials have shown some promising as well as inconclusive results^13^. Recently, its emerging role in regulating vascular homeostasis and cardiovascular disorders has been highlighted^14,15^. Regulation of vascular tone by transcriptional regulation of myosin light chain kinase (MLCK), myosin phosphatase (MYPT)^16,17^, transient receptor potential channel (TRPC6)^18,19^ and soluble guanylate cyclase (sGC)^20^ by Notch has been reported. In addition, Notch is documented to modulate several inflammatory conditions including sepsis and endotoxaemia^21,23^. However, in most of these studies the involvement of Notch was shown in inflammatory cells like macrophages^21,23^, but their regulatory role in vascular function especially during sepsis remains largely unknown. Thus we evaluated the effect of sepsis on vascular Notch signalling and associated vascular dysfunction.

## Materials and methods

### Experimental animals

Adult male Swiss albino mice were procured from All India Institute of Medical Sciences (AIIMS), New Delhi, India. Animals were kept in polypropylene cages in the Departmental Laboratory Animal House (room temperature: 23–25 °C, humidity: 50% and 12–12 h light–dark cycle). Animals had free access to pelleted feed (Ashirwad Industries, Mohali, Punjab, India) and clean drinking water. After an acclimatization period of 15 days, animals weighing around 30–35 g were selected for taking up the study. Study was undertaken following approval of the Institutional Animal Ethics Committee (Approval No: IAEC/19-2/1 via letter No. 127/IAEC/19 Dated 25.10.2019). All methods were performed in accordance with the relevant guidelines and regulations. Further, the study is reported in accordance with ARRIVE guidelines.

### Drugs and chemicals

Nor-adrenaline (NA) and N-[N-(3,5-Difluorophenacetyl)-l-alanyl]-S-phenylglycine t-butyl ester (DAPT) were purchased from Sigma (St. Louis, Mo, USA) while 1400 W dihydrochloride was procured from Cayman (Ann Arbor, Michigan). All other used chemicals/reagents were of analytical grade. Nor-adrenaline was dissolved in 0.1 N hydrochloric acid and DAPT was dissolved in dimethyl sulfoxide (DMSO). All other chemicals were dissolved in distilled water. Concentration of the vehicle (DMSO) used had no significant effect of its own on vascular reactivity.

### Induction of sepsis

Sepsis in mice was induced by ceacal ligation and puncture method (CLP) as described earlier^24^. Briefly, mice were fasted overnight before surgery but allowed water ad libitum. Under xylazine (10 mg/kg b.wt, *i.p*) and ketamine (80 mg/kg b.wt, *i.p.*) anesthesia, a 2 cm ventral midline incision was performed in mice, and the cecum was exposed and then ligated with 2–0 silk just distal to the ileocecal valve to avoid the intestinal obstruction. The caecum was then punctured twice with a 21 G needle and returned to the abdomen. The abdominal incision was then closed in layers. To prevent dehydration, isotonic sodium chloride solution (1 mL/mouse) was injected subcutaneously to all mice. Sham-operated (SO) mice had undergone same surgical procedure except CLP. After induction of sepsis, animals were kept under a close observation for 72 h. The degree of sepsis was assessed by the presence of conjunctivitis, absence of grooming activities with resulting ruffled fur, reduced feed and water intake and lethargy. All the surgical maneuvers were carried out according to the procedures laid down by the Institutional Animal Ethics Committee (IAEC).

### Quantitative reverse transcriptase polymerase chain reaction (RT-qPCR)

At 19 ± 1 h post-surgery, mice were sacrificed under xylazine-ketamine anesthesia and thoracic aortas were collected aseptically. Following removing the adjacent fat and connective tissues, RNA was isolated using commercially available kit (Ambion, USA) by following the manufacturer’s protocol. The samples were treated with RNase-free DNase and DNase was subsequently inactivated by heating at 56 °C for 10 min and immediately chilled at 4 °C. The purity of RNA was checked by biophotometer (Eppendorf, Germany). cDNA synthesis (from 100 ng total RNA) was carried out using Revertaid® First strand cDNA synthesis kit (Thermo Scientific, USA) using Moloney murine leukemia viral reverse transcriptase enzyme by following the manufacturer’s instructions.

RT-qPCR reactions were performed in duplicate using SYBR Green chemistry (PowerUp^TM^SYBR™ Green master mix [2X]; ThermoFischer Scientific, USA) in a realtime thermocycler (QuantStudio 3, Applied Biosystems). Each reaction was consisted of SYBR Green master mix (5 μl), gene-specific forward and reverse primers (0.5 μL each of 10 pmol/μL stock) and cDNA (1 μl) in a total volume of 10 μL. The real-time PCR reaction was started with UDG activation at 50 °C for 2 min, Taq polymerase activation at 95 °C for 2 min followed by 40 cycles of amplification with denaturation at 95 °C for 15 s, annealing (temperature as mentioned in Table 1 for gene specific primer pairs) for 20 s and extension at 72 °C for 1 min each. Primer sequences are listed in Table 1.

**Table 1:** Description of primers.

Gene	Primer sequences	Amplicon size (bp)	Annealing temp. (°C)	References
Notch 1	F5ʹ-GGATCACATGGACCGATTGC-3ʹ R5ʹ-ATCCAAAAGCCGCACGATAT-3ʹ	73	61.5	^25^
Notch 3	F5ʹ-ATGGAGGGCATGGTGGAA-3ʹ R5ʹ-CAAGCTCATCCACTGCATTGA-3ʹ	64	59.0	^25^
DLL4	F5ʹ-GGAACCTTCTCACTCAACATCC-3ʹ R5ʹ-CTCGTCTGTTCGCCAAATCT-3ʹ	141	60.0	^26^
Jag1	F5ʹ-TGGTTGGCTGGGAAATTGA-3ʹ R5ʹ-TGGACACCAGGGCATTC-3ʹ	71	61.5	^25^
Hey1	F5ʹ-CATGAAGAGAGCTCACCCAGA-3ʹ R5ʹ-CGCCGAACTCAAGTTTCC-3ʹ	106	59.6	^26^
MLCK	F5ʹ-AGAAGTCAAGGTAAAGAATGATGT-3ʹ R5ʹ-CGGGTCGCTTTTCATTGC-3	77	60.0	^27^
MYPT1	F5ʹ-AAAGCGACGGTCTACTGGAG-3ʹ R5ʹ-AACAGAATCCGTCTGCGTT-3	118	62.0	^17^
GAPDH	F5ʹ-CCTGCACCACCAACTGCTTAG-3ʹ R5ʹ-GTCTTCTGGGTGGCAGTGATG-3ʹ	109	62.0	^28^

### Western blot analysis

At 19 ± 1 h post-surgery, mice from SO and sepsis group were sacrificed and thoracic aorta were isolated as described above and immediately snap frozen in liquid nitrogen before storing at − 80 °C until further use. The frozen tissues were homogenized in ice-cold radio-immunoassay (RIPA) buffer (Sigma, USA) and the homogenates were then centrifuged at 13,000×*g* for 20 min at 4 °C and protein concentration was determined in the supernatant by Bradford assay kit (Biorad, USA). Equal amount of protein (40 μg) was separated using 12% SDS–polyacrylamide gel electrophoresis (SDS-PAGE) and transferred to polyvinylidene difluoride membrane (PVDF, Milipore). Following blocking the membranes in 5% skimmed milk powder in TBST (w/v) for 2 h at room temperature, the membranes were incubated overnight at 4 °C with the rabbit anti-NICD antibody (1:500, Cell Signaling Technology, Cat no. 4147) or Mouse anti-β-actin (1:5000, Abcam, Cat no. ab170325). After washing, the membranes were further incubated with HRP conjugated goat anti-rabbit IgG (1:2000, Abcam, Cat no. ab6721) or goat anti-mouse (1:2000, Invitrogen, Cat no. 31450). Before incubation with primary and secondary antibodies for respective proteins (NICD and β-actin), the membranes were cut and processed to avoid any cross reactivity between antibodies. The membranes were then developed using SuperSignal West Pico Chemiluminescent Substrate (Thermo Fisher Scientific, USA), and visualized and analysed by iBright (Invitrogen, USA). The intensity of the protein band was expressed in relation to respective β-actin expression.

### Immuno-histochemistry

Thoracic aorta from the mice of SO and CLP groups were fixed in neutral buffered formalin and transverse cross sections (5 μm) from paraffin-embedded aortic tissues were prepared. After rehydration, antigen retrieval in citrate buffer and blocking, the aortic sections were incubation with rabbit anti-mouse polyclonal antibody against cleaved NICD (1:20 dilution, Merck-Milipore) at 4 °C. After that, sections were further incubated with secondary antibody tagged with HRP (goat anti-rabbit polyclonal IgG, 1:100 dilution, Abcam) for 1 h at room temperature in moist chamber. DAB was used as a chromogen and counterstaining was done with Mayer's hematoxylin. Negative control slides were treated similarly except for the incubation with primary antibody. The sections were visualized for NICD protein in mouse aortic section under light microscope (Nikon, Japan). The NICD positive signals were counted in 5 different areas located in four sections from each aorta under 40X magnification. The signal intensity of NICD was measured semi-quantitatively using ImageJ Fiji software and expressed as signal intensity (arbitrary unit).

### DAPT and 1400 W treatment protocol

In vivo administration of γ-secretase inhibitor (DAPT) is reported to reduce inflammation in macrophages and found to be effective in reducing mortality in LPS-induced endotoxaemia^21^, however, its effect on vascular reactivity in unknown. To assess the effect of γ-secretase inhibitor on sepsis-induced vascular hyporeactivity, some mice from SO and/or sepsis groups (n = 6 from each group) were treated with DAPT (@ 40 mg/kg; *i.p*) 1 h before CLP and/or surgery and are grouped as DAPT + SO and DAPT + CLP, respectively. DAPT was dissolved in DMSO (40 mg/ml) and further dilution was made in sterile 0.9% NaCl (1:10) to achieve the final concentration of 4 mg/ml. The dilution of DAPT was prepared afresh just before injection.

iNOS-derived NO plays an important role in vascular hyporeactivity in sepsis and down-regulation of Notch by nitration of tyrosine residues of Notch1 protein in rat alveolar macrophages is reported earlier^29^. Thus to assess the effect of in vivo iNOS inhibition on expression of Notch receptors and its downstream effectors, some septic mice (n = 4) were treated with 1400 W (@1 mg/kg; *s.c*) at 30 min before and 6 and 12 h post-CLP in a separate set of experiment.

### Functional studies by Wire Myography

Mice from different treatment groups (SO, CLP, DAPT + SO and DAPT + CLP) were sacrificed by bleeding from vena cava under xylazine-ketamine anesthesia at 19 ± 1 h post-surgery. Thorax and abdomen were cut open and lungs and heart were taken out en-bloc along with the thoracic aorta and immediately placed in ice cold (4 °C) Modified Krebs–Henseleit solution (MKHS) with the composition (mmol/L): 118.0 NaCl, 4.7 KCl, 2.5 CaCl_2_·2H_2_O, 1.2 MgSO_4_·7H_2_O, 1.2 KH_2_PO_4_, 11.9 NaHCO_3_ and 11.1 d-glucose. Thoracic aorta was cleaned off the adhering connective tissues under dissection stereo microscope (Motic, China) and aortic rings of 3–4 mm length were prepared without damaging the underlying endothelial layer. Arterial rings were mounted with the help of stainless steel wire (40 µm) and equilibrated under a resting tension of 4 mN in the thermostatically controlled (37 ± 1 °C) wire myograph (DMT, Denmark) of 5 ml capacity containing MKHS continuously bubbled with medical gas (74% N_2_ + 21% O_2_ + 5% CO_2_). The isometric tensions were recorded with the help of LabChart Pro V7 software (ADInstruments, Australia). Before performing any experimental protocol, tissue viability was assessed by recording the contractile response of aortic rings to high K^+^ (80 mM) depolarizing solution.

Nor-adrenaline (NA) is commonly used vasopressor agents in septic patient. Thus to evaluate the effect of sepsis and DAPT pre-treatment on vascular reactivity to NA, concentration-dependent (0.1 nM–10 µM) contractile response to NA was recorded in the arterial rings of the mice from different groups at an increment of 0.5 log concentration unit. Calcium chloride (CaCl_2_)-induced contraction in K^+^-depolarized vascular tissue preparations is a standard protocol for the functional assessment of the voltage-dependent L-type calcium channels and receptor-independent calcium-dependent contraction (preferably myosin light chain kinase-dependent contraction). To assess the effect of DAPT pre-treatment on receptor-independent vascular contraction, cumulative concentration responses of CaCl_2_ were recorded in the arterial rings from different groups of mice. In this set of experiment, following equilibration period, the aortic rings were contracted with 80 mM K^+^-depolarizing solution followed by two to three washing with Ca^2+^-free MKHS with EGTA solution to remove any extracellular calcium. Then the tissue was incubated with Ca^2+^-free K^+^-depolarizing solution without EGTA for 30 min. Then concentration-dependent contractions to CaCl_2_ (10 μM–10 mM) were elicited at an increment of 0.5 log concentration unit.

### Statistical analysis

Results are expressed as mean ± SEM. ‘n’ refers to the number of animals used in each experimental protocol. The difference in survival rates was determined by Kaplan–Meier test followed by log-rank test.

The efficiencies of respective primer sets were determined by detecting the C_T_ value of different dilutions of cDNA samples and plotting the linear regression between observed C_T_ value vs. log concentration of cDNA. Efficiencies of all the primer sets were calculated and found to be between 89 and 102%. Data were then corrected for efficiencies for each primer followed by normalization for expression of the target genes to that of the reference gene (GAPDH). The relative change in gene expression was calculated using the formula as mentioned earlier^30^.

The E_max_ (the maximal response) and EC_50_ (the concentration producing 50% of the maximal response) values were determined by nonlinear regression analysis using Graph Pad Prism V.4.00 software (San Diego, California). Potency refers to pD_2_ value of the agonist which is equivalent to −log of the EC_50_ value. Mean values of two groups were analyzed by unpaired student’s ‘t’ test whereas means of more than two groups were analyzed by one-way ANOVA followed by Tukey’s post-hoc test. Concentration-dependent agonist response data were analyzed by two-way ANOVA followed by Bonferroni post-hoc test using GraphPad Prism software. Difference in the values was considered statistically significant at *p* < 0.05.

### Ethics approval

Study was undertaken following approval of the Institutional Animal Ethics Committee of Uttar Pradesh Pandit Deen Dayal Upadhyaya Pashu-Chikitsa Vigyan Vishwavidyalaya Evam Go Anusandhan Sanstahan (DUVASU), Mathura, India (Approval No: IAEC/19-2/1 via letter No. 127/IAEC/19 Dated 25.10.2019).

## Results

### Effect on survival time

**Figure 1:**
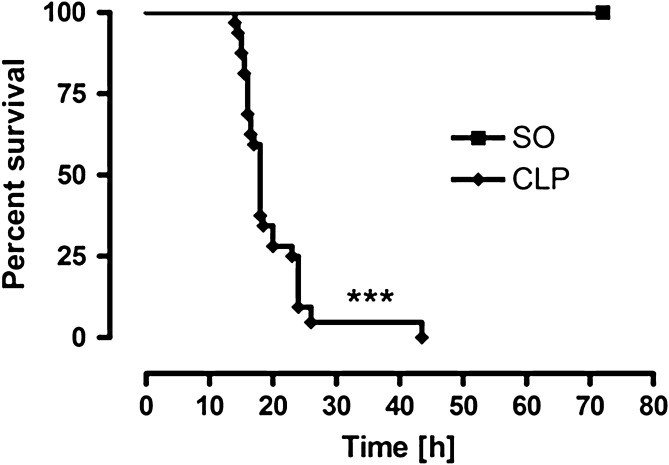
Effect of sepsis on survival time in mice. Sepsis (CLP) significantly (*p* < 0.001) reduced the mean survival time in septic mice (19.97 ± 1.04 h; n = 30) in comparison to the sham-operated (SO) mice. All the SO mice (n = 12) survived during the 72 h observation period. The overall difference in survival rate was determined by the Kaplan–Meier test followed by the log-rank test. *p < 0.001 vs. SO.

Figure 1 shows the survival curves of the mice of septic (CLP) and sham-operated (SO) groups. The mean survival time in septic mice (19.97 ± 1.04 h; n = 30) was significantly lower (*p* < 0.001) as compared to that in SO mice. All the SO mice (n = 12) survived during the observation period of 72 h. Given that, the septic animals were survived for around 20 h, all the aortic samples for the present study were collected at 19 ± 1 h post-CLP or surgery.

### Effect of sepsis on the expression of Notch receptors in mouse aorta

**Figure 2:**
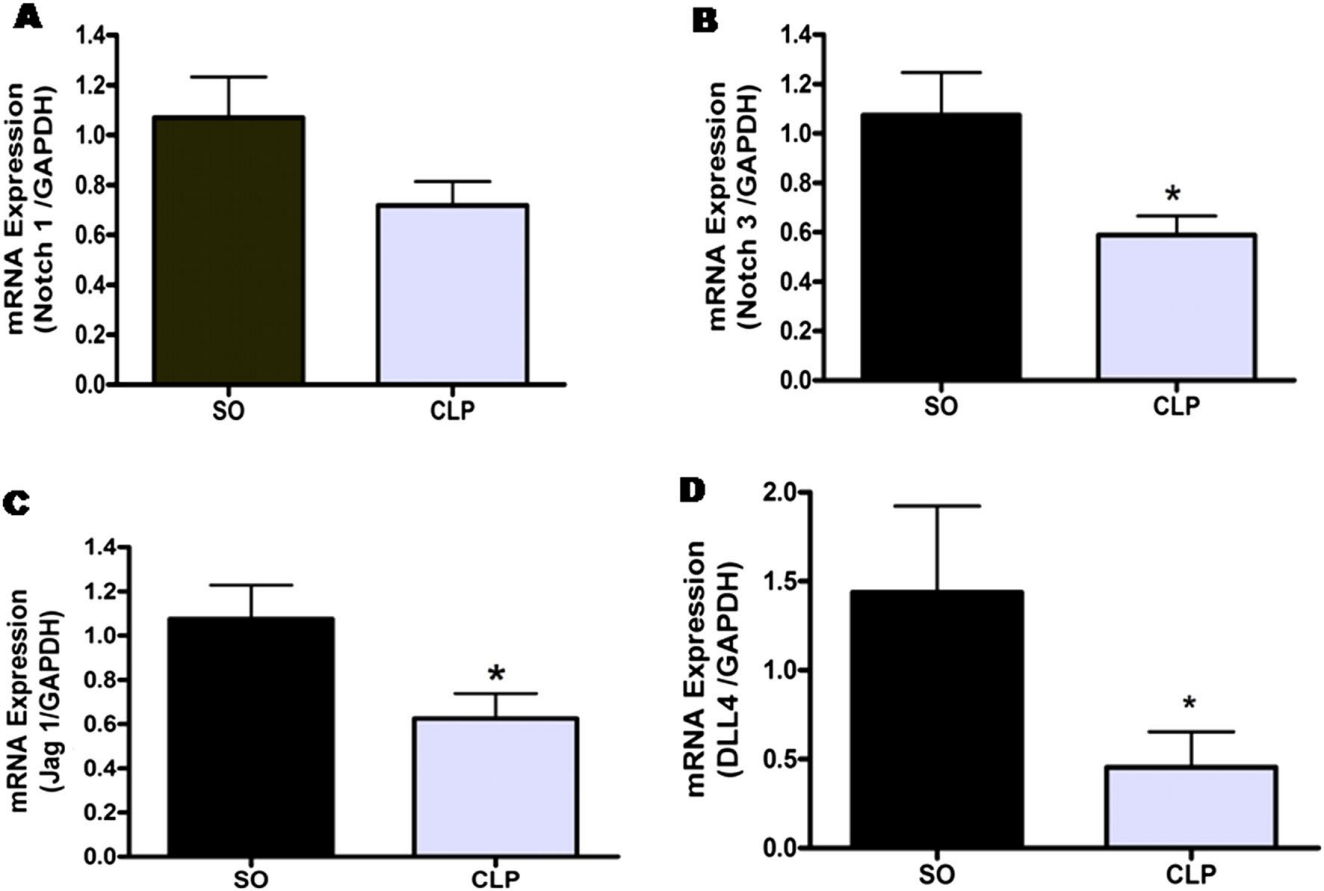
Effect of sepsis on the different components of Notch signaling in mouse aorta. Bar diagram showing the effect of sepsis on comparative mRNA expressions of Notch1 receptor (**A**), Notch3 receptor (**B**), Jag1 (**C**) and Dll4 (**D**) ligands in mouse aorta from sham-operated (SO) and septic (CLP) mice. Data are presented as mean ± SEM; n = 7–8. Vertical bars represent SEM. Data were analyzed by unpaired student’s ‘t’ test. *p < 0.05 vs. SO.

As illustrated in Fig. 2A, sepsis did not produce significant change in Notch1 mRNA transcript expression in mouse aorta, albeit, there was 32.71% decrease in the mRNA expression as compared to SO mice (0.72 ± 0.10 vs. 1.07 ± 0.16, n = 7–8). However, aortic Notch3 mRNA expression was significantly (*p* < 0.05) down-regulated in septic mouse aorta (0.59 ± 0.08, n = 8) in comparison with SO mice (1.07 ± 0.17, n = 7) as shown in Fig. 2B.

### Effect of sepsis on the expression of the Notch ligands in mouse aorta

As illustrated in Fig. 2C, sepsis significantly (*p* < 0.05) attenuated the mRNA expression of Jag1 ligand in mouse aorta as compared to that observed in SO mice (0.62 ± 0.11 vs. 1.08 ± 0.15, n = 7–8). Further, aortic Dll4 mRNA expression (Fig. 2D) was also significantly (*p* < 0.05) down-regulated in septic mouse aorta (0.45 ± 0.20, n = 8) as compared to SO mice (1.44 ± 0.48, n = 7).

### Effect of sepsis on the expression of the Notch intracellular domain (NICD) in mouse aorta

**Figure 3:**
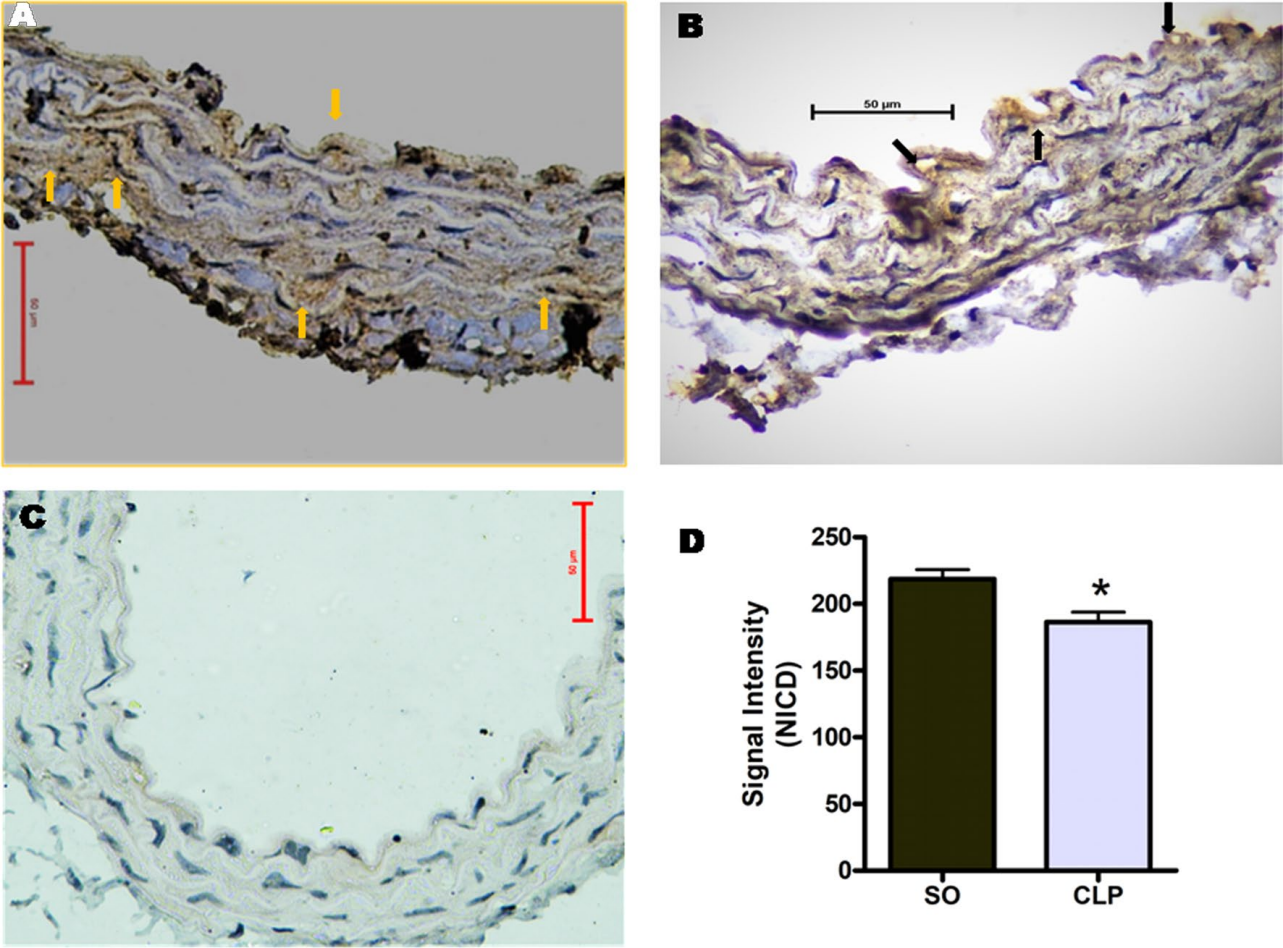
Representative immuno-histochemistry images of Notch intracellular domain (NICD) on aortic sections from sham-operated (SO; **A**) and septic (CLP; **B**) mice, and the comparative signal intensity (arbitrary unit) of NICD (**D**). (**C**) Represents negative control (without primary antibody). Horizontal bar represents 50 μm scale bar; IHC, 400 ×. Arrows show positive signal. NICD positive signals were counted in 5 different areas located in four sections. Data are presented as mean ± SEM, n = 4. Vertical bars represent SEM. Data were analyzed by unpaired student’s ‘t’ test. *p < 0.05 vs. SO.

Functional activity of Notch receptor relies on the release of cleaved Notch intracellular domain (NICD) from full length receptor and its binding to the transcriptional factor for transcriptional regulation. Thus to relate the change in mRNA expression to the activity of Notch receptors, the protein level of activated (cleaved) NICD was evaluated in the mouse aorta from different groups by immuno-histochemistry (IHC). As illustrated in the representative images (Fig. 3A,B) and summarized in Fig. 3D, the signal intensity of immunoreactive NICD was significantly (*p* < 0.05) lesser in aortic tissue sections from CLP group as compared to those observed in SO mice. Negative control slides did not show any positive signal to NICD (Fig. 3C).

**Figure 4:**
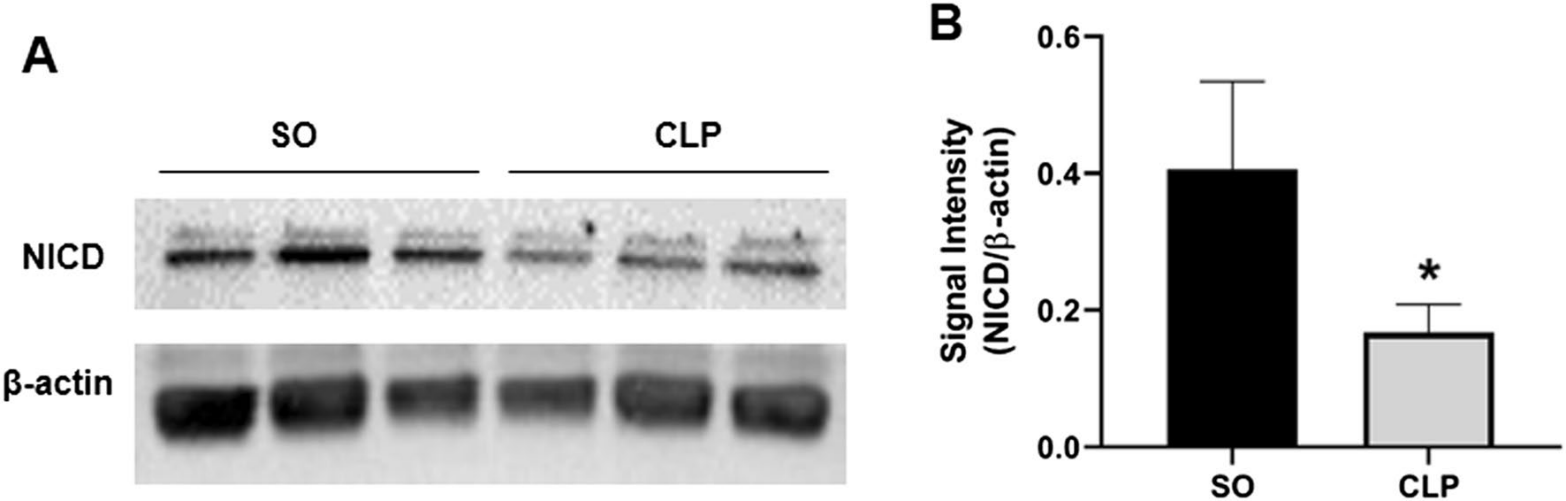
Sepsis down-regulated Notch intracellular domain (NICD) level in mouse aorta. Representative Western blot images (**A**) and bar diagram (**B**) showing the effect of sepsis on the protein level of NICD in mouse aorta (n = 5). Vertical bars represent SEM. ‘n’ represents number of independent samples from different groups. Data were analyzed by unpaired student’s ‘t’ test. *p < 0.05 vs. SO. The NICD and β-actin bands were cropped from full blot (Supplementary Fig. S1 online).

Further, the protein levels of NICD in the mouse aorta from SO and CLP mice were compared using Western blot. As illustrated in Fig. 4, as compared to that observed in SO mice, sepsis significantly (*p* < 0.05) down-regulated the NICD level in mouse aorta (0.41 ± 0.06 vs. 0.17 ± 0.02; n = 5).

### Effect of sepsis on mRNA expression of the Notch downstream effector genes

**Figure 5:**
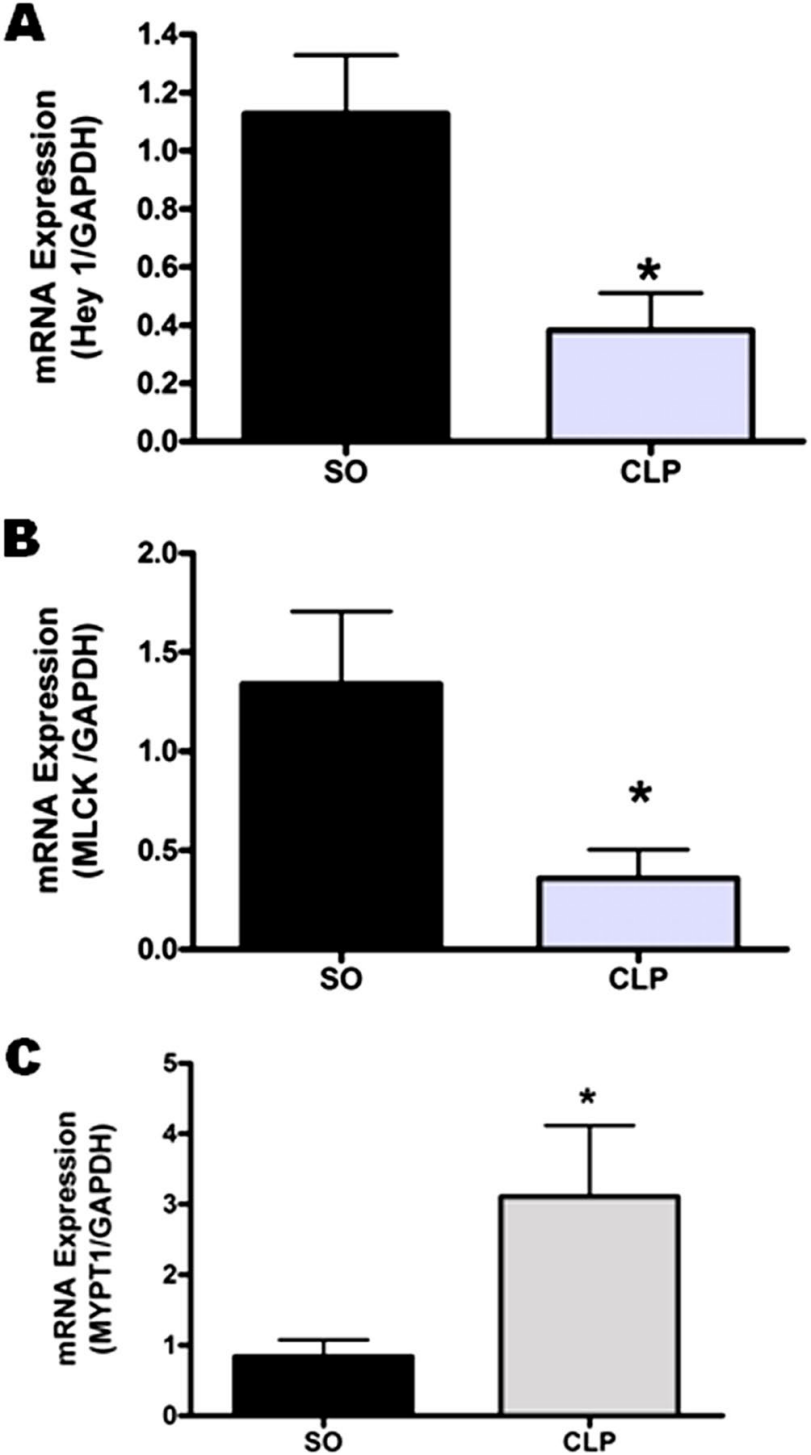
Effect of sepsis on the expression of Notch downstream effector genes in mouse aorta. Bar diagrams showing the effect of sepsis on mRNA expression of Hey1 (**A**; n = 7–8), myosin light chain kinase (MLCK; **B**; n = 7) and myosin phosphatase (MYPT1; **C**; n = 4) in mouse aorta. *SO* sham-operated, *CLP* sepsis. Data are presented as mean ± SEM. Vertical bars represent SEM. Data were analyzed by unpaired student’s ‘t’ test. *p < 0.05 vs. SO.

Activation of the Notch signalling results in transcriptional promotion of different effector genes among which hairy/enhancer of split-related proteins (Hey1) is one of the most studied downstream effector genes. Thus we evaluated the aortic mRNA expression of Hey1 in sepsis. As shown in Fig. 5A, sepsis significantly (*p* < 0.05) attenuated the mRNA expression of Hey1 in mouse aorta as compared to that observed in the mice of SO group (0.38 ± 0.13 vs. 1.13 ± 0.20, n = 7–8).

Notch signalling is reported to regulate the expression of myosin light chain kinase (MLCK) and myosin phosphatase targeting subunit-1 (MYPT1) in vascular beds. Thus we evaluated the effect of sepsis on the mRNA expression of both these enzymes. Sepsis significantly (*p* < 0.05) attenuated the mRNA expression of MLCK (Fig. 5B) in mouse aorta (0.36 ± 0.14 vs. 1.34 ± 0.37, n = 7) while MYPT1 expression (Fig. 5C) was significantly (*p* < 0.05) increased (3.11 ± 1.01 vs. 0.84 ± 0.24, n = 4) as compared to that observed in SO group.

### Effect of pre-treatment with γ-secretase inhibitor (DAPT) on mRNA expression of gene related to Notch signalling

**Figure 6:**
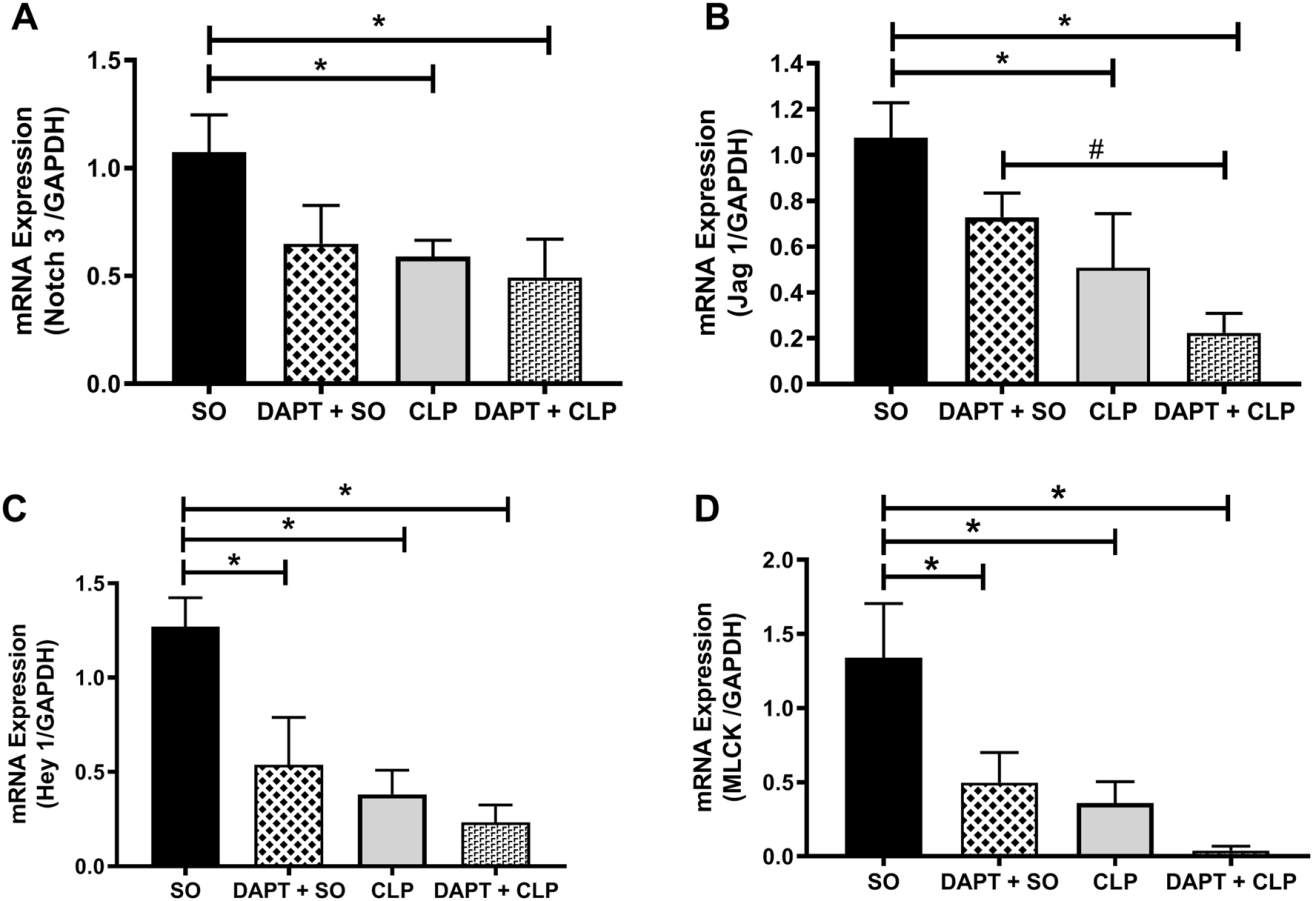
Effect of DAPT treatment on comparative aortic Notch signaling and its effector genes. Bar diagrams showing the effect of in vivo treatment with DAPT, a selective inhibitor of γ-secretase, on mRNA expression of Notch3 receptor (**A**), Jag1 ligand (**B**), Hey1 (**C**) and MLCK (**D**) genes in the aorta of SO and CLP mice. *SO* sham-operated, *CLP* sepsis. Data are presented as mean ± SEM. Vertical bars represent SEM; n = 7–8. Data were analyzed by one-way ANOVA followed by Tukey’s post-hoc test. *p < 0.05 vs. SO, ^#^*p* < 0.05 vs. DAPT + SO.

Figure 6 illustrates the effect of DAPT pre-treatment on comparative aortic mRNA expression profiles of Notch3 receptor, Jag1 ligand and the effector genes viz*.* Hey1 and MLCK in SO and CLP mice. As shown in Fig. 6A, pre-treatment with DAPT did not produce any significant change in the aortic Notch3 expression either in SO (0.65 ± 0.07 vs. 1.07 ± 0.17, n = 7) or septic (0.49 ± 0.06 vs. 0.59 ± 0.08, n = 8) mice in comparison to their respective untreated control. Similarly, no significant change was observed in the aortic Jag1 expression in SO and septic mice following DAPT treatment as compared to their respective control (Fig. 6B). However, aortic expression of both Notch3 receptor and Jag1 ligand were found to be significantly (*p* < 0.05) lower in CLP and DAPT + CLP groups as compared to SO mice.

We then evaluated the expression profiles of downstream effector genes of notch signalling in mouse aorta from SO and CLP mice following DAPT treatment. Interestingly, as illustrated in Fig. 6C,D, DAPT treatment in SO mice significantly (*p* < 0.05) reduced the aortic mRNA expression of Hey1 (0.54 ± 0.11, n = 5) as well as MLCK (0.50 ± 0.08, n = 6) in comparison to untreated control (1.27 ± 0.15 and 1.34 ± 0.37; n = 7, respectively). Similarly, comparatively 39.47% (0.23 ± 0.03 vs. 0.38 ± 0.12, n = 7–10) and 89.89% (0.04 ± 0.01 vs. 0.36 ± 0.14, n = 8) reduction in the expression of Hey1 and MLCK, respectively, were observed in the aorta of septic mice following DAPT treatment (Fig. 6C,D).

### Effect of pre-treatment with DAPT on vascular reactivity in septic mice

#### Effect on high K^+^ (80 mM) depolarizing solution-induced contraction

**Figure 7:**
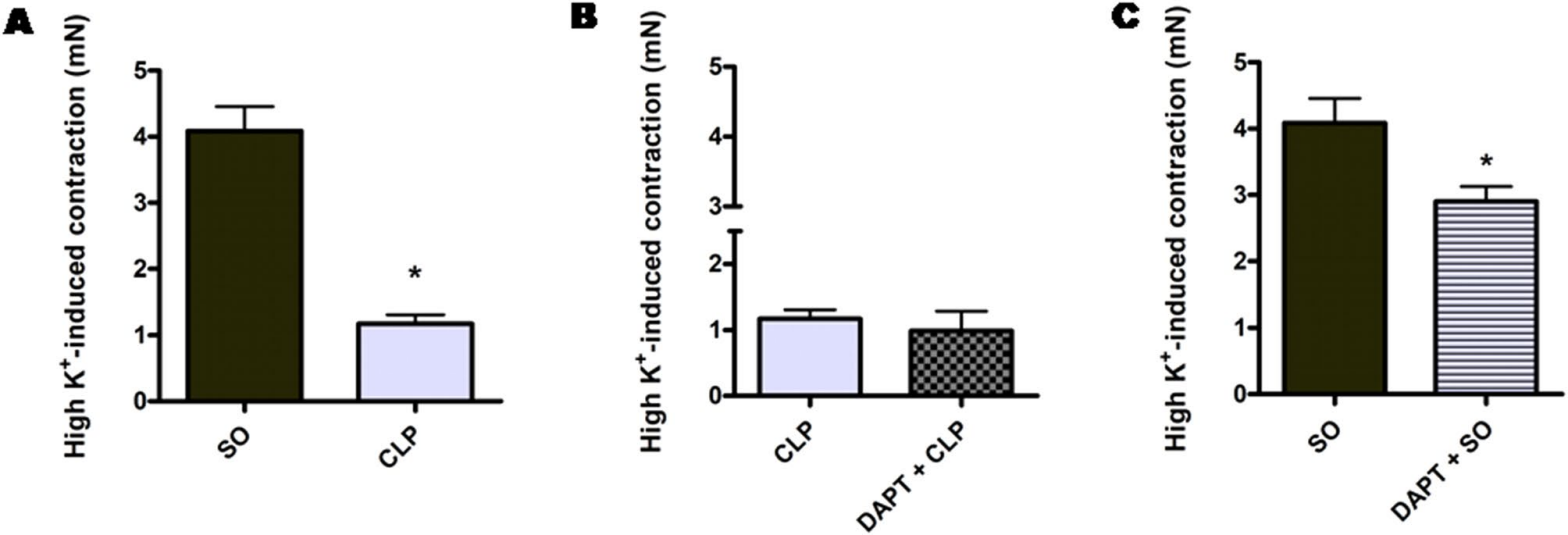
Effect of DAPT pre-treatment on high K^+^ (80 mM) depolarizing solution-induced contraction in aorta of septic mice. Bar diagrams (**A**–**C**) showing the comparative maximum contraction elicited by high K^+^ (80 mM) solution in the aortic rings isolated from sham-operated (SO), sepsis (CLP), DAPT pre-treated sepsis (DAPT + CLP) and DAPT pre-treated SO (DAPT + SO) groups. Data are presented as mean ± SEM; n = 6. Vertical bars represent SEM. Data were analyzed by unpaired student’s ‘t’ test. *p < 0.05 in comparison to SO.

Figure 7 depicts the effect of DAPT pre-treatment on high K^+^ (80 mM) depolarizing solution (KDS)-induced contraction in aortic rings from different groups. The aortic rings from SO mice developed sustained contraction (4.09 ± 0.37 mN, n = 10) to KDS. Compared to SO mice, sepsis significantly (*p* < 0.05) reduced the contraction to 1.17 ± 0.14 mN (n = 10). Pre-treatment of septic mice with DAPT did not result in any improvement in sepsis-induced attenuated aortic response to KDS (0.99 ± 0.30 mN vs. 1.17 ± 0.14 mN, n = 10). However, in the aortic rings from SO mice pre-treated with DAPT, significant (*p* < 0.05) reduction in high K^+^-induced contraction (2.90 ± 0.22 mN, n = 10) was observed as compared to SO mice (4.09 ± 0.37 mN, n = 10).

#### Effect on nor-adrenaline-induced aortic contraction

**Figure 8:**
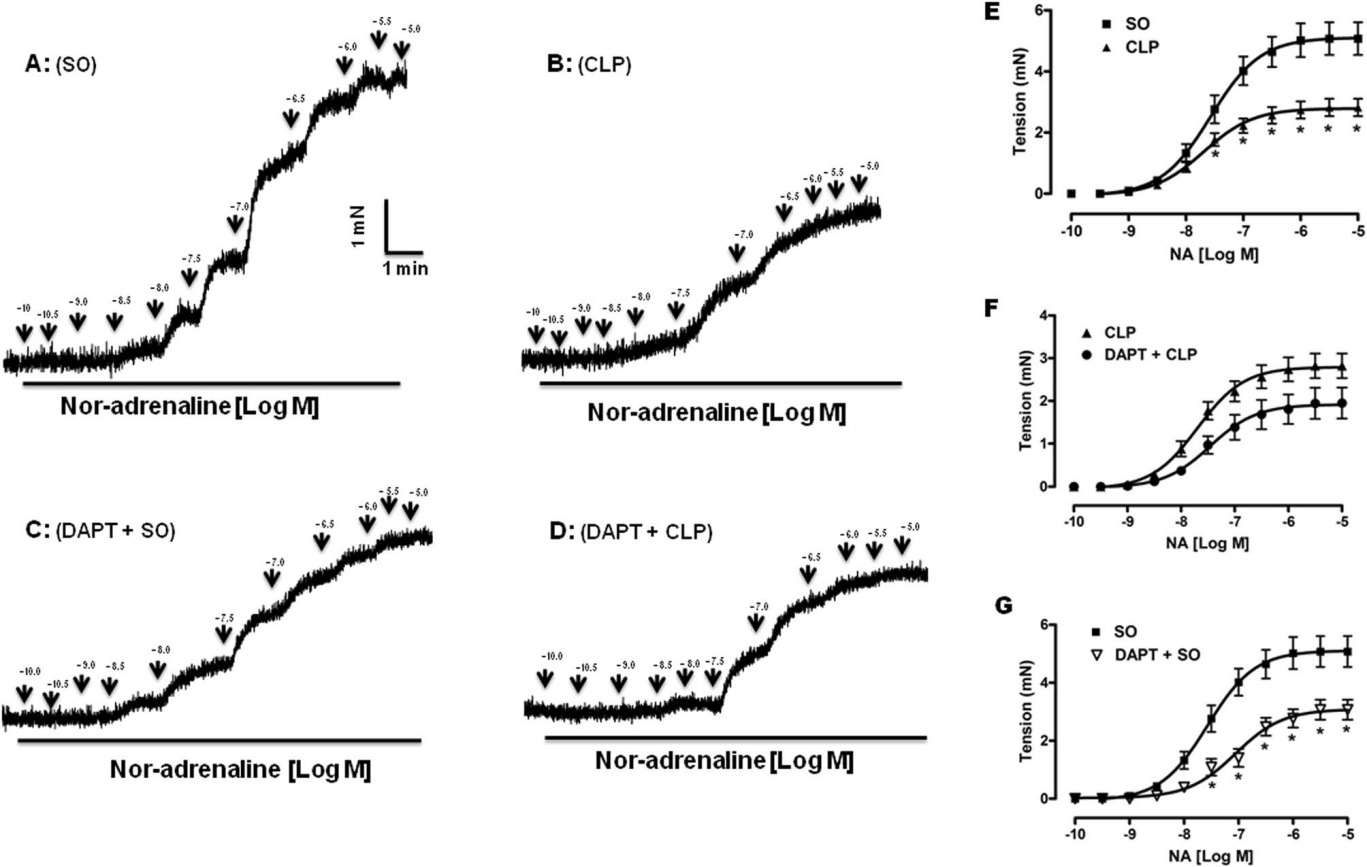
Effect of DAPT pre-treatment on hyporeactivity of the aortic ring to nor-adrenaline (NA) in septic mice. Representative tracings show the concentration-dependent contractile response to NA (0.1 nM–1 µM) in the aorta from sham-operated (SO; **A**), sepsis (CLP; **B**), DAPT pre-treated SO (DAPT + SO; **C**) and DAPT pre-treated sepsis (DAPT + CLP; **D**) groups. Comparative line diagrams (**E**–**G**) showing the mean concentration–response curves of NA from different groups. Data are presented as mean ± SEM; n = 6. Vertical bars represent SEM. Data were analyzed by two-way ANOVA followed by Bonferroni post-hoc tests. *p < 0.05 in comparison to SO.

Figure 8 illustrates the reactivity of mouse aorta to nor-adrenaline (NA) in different groups. The representative tracings (8A) and line diagrams (8E) of cumulative concentration–response to NA show that aortic rings from SO mice exhibited concentration-dependent (0.1 nM–1 µM) contractions to NA. The pD_2_ and E_max_ values were 7.57 ± 0.11 and 5.08 ± 0.53 mN (n = 6), respectively. As illustrated in the Fig. 8B,E, sepsis significantly (*p* < 0.05) decreased the contractile responses to NA (E_max_ 2.82 ± 0.29 mN; n = 6) without affecting the potency of the agonist (pD_2_: 7.69 ± 0.11; n = 6). The tracings in Fig. 8C,D show the concentration-dependent contractile effect of NA on the aortic rings from CLP and SO mice pre-treated with DAPT, respectively. As shown in Fig. 8F, pre-treatment with DAPT in septic mice (DAPT + CLP) did not produce any improvement in CLP-induced attenuation of aortic reactivity to NA, rather there was 30.85% decrease in the maximal effect of NA as compared to untreated CLP group. However, as compared to SO alone, DAPT pre-treatment in SO mice significantly (*p* < 0.05) shifted the concentration–response curve of NA towards right (Fig. 8G) with significant (*p* < 0.05) decrease in maximum aortic response (E_max_: 3.07 ± 0.34 mN vs. 5.08 ± 0.53 mN) and potency (pD_2_: 7.06 ± 0.12 vs. 7.57 ± 0.11, n = 6).

#### Effect on CaCl_2_-induced aortic contraction

**Figure 9:**
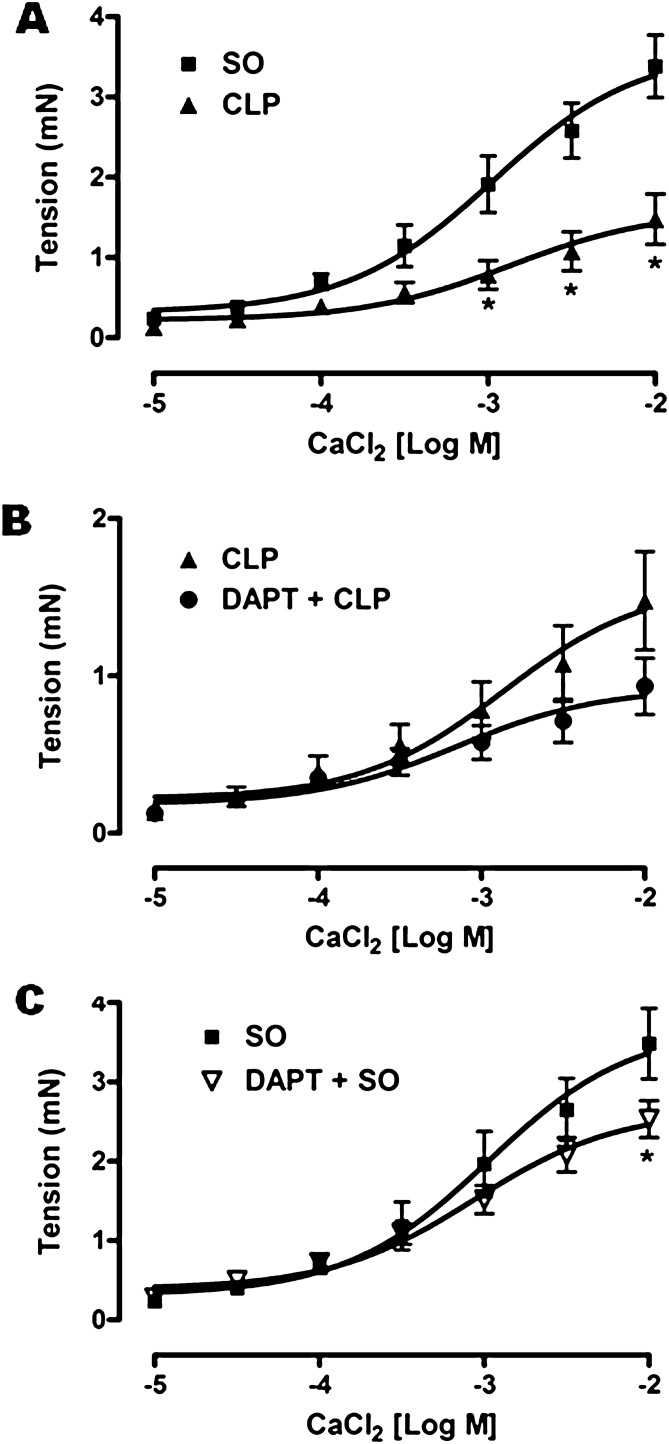
Effect of DAPT pre-treatment on hyporeactivity of the aortic ring to calcium-chloride (CaCl_2_) in septic mice. Comparative line diagrams (**A**–**C**) showing the mean concentration–response curves of CaCl_2_ on the aorta isolated from sham-operated (SO), sepsis (CLP), DAPT pre-treated sepsis (DAPT + CLP) and DAPT pre-treated SO (DAPT + SO) groups. Data are presented as mean ± SEM; n = 6. Vertical bars represent SEM. Data were analyzed by two-way ANOVA followed by Bonferroni post-hoc tests. *p < 0.05 in comparison to SO.

The aortic rings from SO mice exhibited concentration-dependent (10 μM–1 mM) contractions to CaCl_2_ with pD_2_ and E_max_ values of 2.97 ± 0.18 and 3.38 ± 0.38 mN (n = 6), respectively. As illustrated in Fig. 9A, sepsis significantly (*p* < 0.05) decreased the maximal response (E_max_: 1.48 ± 0.31 mN; n = 6) along with potency (pD_2_: 2.87 ± 0.30; n = 6) of CaCl_2_. Pre-treatment with DAPT in septic mice did not produce any improvement in the attenuated aortic response to CaCl_2_ in comparison to untreated CLP group (E_max_: 0.93 ± 0.18 mN, pD_2_: 3.15 ± 0.30; n = 6), rather there was 37.16% decrease in the maximal effect of CaCl_2_ (Fig. 9B). However, as shown in Fig. 9C, maximum response to CaCl_2_ was significantly (*p* < 0.05) attenuated in the aortic rings from DAPT pre-treated SO mice (2.53 ± 0.23 mN, n = 6) as compared to untreated SO group (3.38 ± 0.38 mN, n = 6), albeit there was no significant change in the potency (3.06 ± 0.15 vs. 2.97 ± 0.18, n = 6).

### Effect of in vivo inhibition of iNOS on mRNA expressions of genes related to Notch signalling

**Figure 10:**
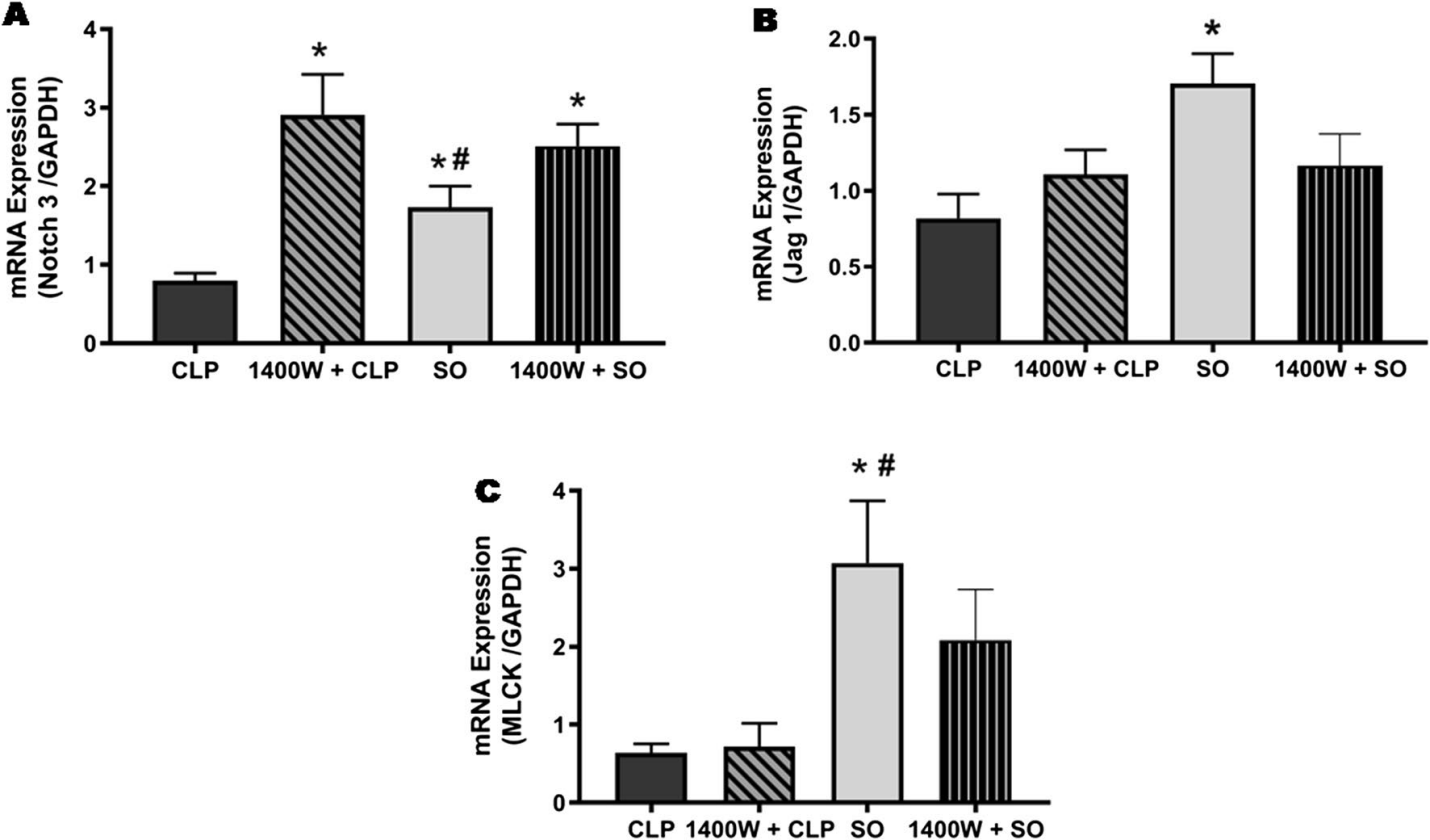
Role of iNOS/NO pathway in sepsis-induced attenuation of Notch signaling. Bar diagrams showing the effect of in vivo treatment with 1400 W, a selective inhibitor of inducible nitric oxide synthase (iNOS), on sepsis-induced attenuation of mRNA expression of Notch3 receptor (**A**), Jag1 ligand (**B**) and MLCK (**C**) genes in mouse aorta. *SO* sham-operated, *CLP* sepsis. Data are presented as mean ± SEM. Vertical bars represent SEM; n = 4–7. Data were analyzed by one-way ANOVA followed by Tukey’s post-hoc test. *p < 0.05 vs. CLP; ^**#**^*p* < 0.05 vs. 1400 W + CLP.

As we observed significant (*p* < 0.05) decrease in mRNA expression of Notch3 receptor, Jag1 ligand and myosin light chain kinase (MLCK) in the aorta of septic mice, thus to evaluate the role of inducible nitric oxide synthase (iNOS)-derived NO (being an inflammatory mediator), in down-regulation of these genes, if any, septic mice were treated with 1400 W, a specific inhibitor of iNOS. The septic animals (n = 4) treated with 1400 W appeared to be dull, depressed and in almost morbid stage at 19 ± 1 h post-CLP as seen in case of septic control mice as well. As shown in Fig. 10A, following in vivo inhibition of iNOS/NO pathway by 1400 W significant (*p* < 0.05) increase in the mRNA expression of Notch3 was observed in the aorta of septic mice (2.91 ± 0.51, n = 4) as compared to untreated CLP group (0.80 ± 0.10, n = 7). Interestingly, the mRNA expression level of aortic Notch3 following 1400 W treatment in CLP mice was found to be even significantly (*p* < 0.05) higher than SO mice (1.73 ± 0.26, n = 6). However, 1400 W treatment in septic mice did not produce any significant alteration in mRNA expression of Jag1 in the aorta of septic mice (1.11 ± 0.16, n = 4), though there was 35.37% increase in the expression (Fig. 10B) as compared to that observed in untreated CLP group (0.82 ± 0.16, n = 7). Similarly, down-expression of MLCK in septic mouse was also remained unaltered following 1400 W treatment in septic mice (0.72 ± 0.30 vs. 0.88 ± 0.26, n = 4–7) as compared to untreated CLP group (Fig. 10C). Treatment with 1400 W did not produce any significant change in the mRNA expression of Notch3 (2.51 ± 0.14 vs. 1.73 ± 0.26; n = 6), Jag1 (1.17 ± 0.10 vs. 1.71 ± 0.20; n = 6) and/or MLCK (2.09 ± 0.32 vs. 3.07 ± 0.80; n = 6) in SO mice as compared to untreated SO group (Fig. 10A–C).

## Discussion

Major findings of the present study were (1) Notch3 was significantly down-regulated in mouse aorta following CLP, (2) Down-regulation of aortic Notch signalling in sepsis was associated with corresponding decrease in aortic MLCK and increase in MYPT1 expression, (3) pre-treatment with DAPT did not improve sepsis-induced vascular hyporeactivity to NA or CaCl_2_, rather significantly attenuated the response to these vasoconstrictors in SO mice, (4) treatment with 1400 W reversed attenuated Notch3 expression without significantly altering Jag1 and MLCK expression in septic mouse aorta.

The evolutionary conserved Notch signalling pathway is operational in both vascular smooth muscle (VSMC) and endothelial cells (EC)^11^. Vascular smooth muscle cells mainly express Notch1 and 3 receptors while Notch1 and 4 receptors are present in endothelium^31,32^. Among the Notch ligands, Dll4 is exclusively expressed in the endothelium while Jag1 is present in both EC and VSMC^31,33^. In the present study, we observed significant (*p* < 0.05) decrease in the mRNA expression of Notch3 receptor while a marked (though non-significant) decrease in mRNA (32.71%) expressions of Notch1 receptor in septic mouse aorta as compared to SO mice. Further, we evaluated the level of NICD (activated or cleaved Notch1) to assess the functional consequence of sepsis-induced change in mRNA expression of Notch1 receptor at the level of downstream pathway activity. Sepsis significantly down-regulated the protein level of NICD in mouse aorta as compared to SO control.

Further, the aortic mRNA expressions of both Jag1 and Dll4 ligands were also significantly (*p* < 0.05) down-regulated in sepsis. Downstream to the receptor-ligand interaction, mRNA expression of an effector gene (Hey1) was also significantly (*p* < 0.05) attenuated in septic mouse aorta suggesting an overall decrease in Notch signalling in mouse aorta following polymicrobial sepsis. In contrast to our observation of sepsis-induced down-regulation of Notch in mouse aorta, activation of Notch pathway either in peritoneal macrophage of endotoxaemic rats or in LPS-stimulated macrophage cell lines was reported earlier^21^.

Excessive release of pro-inflammatory cytokines occurs in sepsis^34^ which are mainly produced by macrophages and helper T cells, however, cytokines released form VSMC (TNF-α, IL-1α and IL-1β) and EC (IL-1α, IL-1β) also plays important role in regulating vascular tone^35,36^. Both IL-1β and TNF-α can suppress expression and activity of Notch3 in VSMC^37,38^. Although we did not estimate circulating levels of cytokines, but in view of the established cytokine storm in sepsis, involvement of these pro-inflammatory cytokines in down-regulating Notch signalling in septic mouse aorta in the present study cannot be ruled out. The complex cross-talk between NF-κB and Notch has also revealed that Notch can either stimulate or inhibit NF-κB signalling^39,40^. Therefore, role of NF-κB activation during sepsis-induced suppression of aortic Notch signalling needs further investigation.

Force of contraction in VSMC is determined by the opposing actions of MLCK and myosin phosphatase (MP), and MLCK is shown to regulate blood pressure under patho-physiological conditions^41,42^. Vasoconstriction is triggered by Ca^2+^/calmodulin-dependent activation of MLCK following entry of Ca^2+^ through voltage-sensitive Ca^2+^ channels or via IP3-mediated release of Ca^2+^ from sarco-endoplasmic reticulum (SER). In addition, smooth muscle contraction is also facilitated by ‘Ca^2+^ sensitization pathway’ following inactivation of MP through phosphorylation of MP regulatory subunit, myosin phosphatase targeting subunit-1 (MYPT1), by Rho-kinase^43,44^. Identification of Notch-responsive element within MLCK promoter region that binds Notch receptor complex has provided the evidence for Notch-dependent transcriptional regulation of contractile genes of VSMC^16^. Additionally, stimulation of Notch receptors in primary aortic SMCs by Jag1 ligand increased the endogenous MLCK transcript with corresponding induction of Notch target gene^16^. Further, Jag1 has also been shown to selectively induce MLCK gene expression and inhibit MP function in a Rho kinase-dependent manner, albeit, the exact mechanism of regulation of Rho kinase/MYPT1/MP by Notch signalling is yet to be established^17^. In the present study, we observed significant (*p* < 0.05) reduction in MLCK and rise in MYPT1 expressions in septic mouse aorta with corresponding down-regulation of Notch receptors and its effector gene. Further, results from vascular reactivity study showed significant (*p* < 0.05) decrease in aortic response to both NA (receptor-dependent contraction) and CaCl_2_ (receptor-independent contraction) where MLCK activation is a penultimate step during arterial force generation^45^. Consistent to our findings, it has been reported that although the total MYPT1 level remain elevated, the phosphorylated MYPT1 were significantly reduced in the mesenteric artery from endotoxaemic rats through cGMP-dependent manner thus indicating that despite of the compensatory up-regulation of the component of Ca^2+^ sensitization pathway, the signalling molecules remain poorly phosphorylated resulting in activation of myosin phosphates leading to reduced vascular reactivity in endotoxamic rats^46^. Additionally, knocking down of Notch3 showed impaired myogenic response in caudal, cerebral and renal arteries due to reduced activation of MLC and RhoA/Rho kinase signalling in mice^47–49^. Earlier we have reported that down-regulation and desensitization of aortic α_1D_-adrenoceptor is one of the underlying mechanisms for reduction in arterial response to NA in sepsis^8^. Given that Notch can transcriptionally regulate different receptors/ion channels and downstream effector genes essential for vascular response^19,20,50^, evaluating the role of Notch in regulation of vascular α_1D_-adrenoceptor expression may lead to novel therapies to target sepsis-induced ‘vasoplegia’.

Functional role of Notch in controlling vascular response was established from the finding that mutation in human Notch3 receptor caused CADASIL (cerebral autosomal dominant arteriopathy with subcortical infarcts and leukoencephalopathy) syndrome^51^. Additionally, inhibition of smooth muscle specific Notch signalling showed reduced blood pressure response to vasoconstrictors^16^. Consistent to these reports, we also observed significant decrease in vascular reactivity to NA, CaCl_2_ and high K^+^-depolarizing solution in aortic rings from SO mice pre-treated with DAPT, as compared to untreated SO mice suggesting that basal level of Notch signalling and its activation are required for arterial force generation. This reduced vascular reactivity to contractile agents was further supported by the notion that the aortic mRNA expression of MLCK in SO mice was significantly down-expressed following DAPT treatment as compared to the untreated control group. Thus systemic blockade of Notch signalling by DAPT did not improve sepsis-induced vascular hyporeactivity to contractile agents rather there was further decrease in the aortic smooth muscle response to NA (by 30.85%), CaCl_2_ (by 37.16%) and high K^+^ (by 15.38%) as compared to that observed in untreated septic mice. In consistent to these findings, we also observed a marked reduction (89.89%) in the aortic MLCK expression in the septic mice following DAPT treatment in comparison to the untreated CLP mice. Similarly, mutant mice expressing a dominant-negative Mastermind-like protein (SM22-Cre^+^/DNMAML1^+^) also showed reduced reactivity to phenylephrine (another *alpha*_1_ agonist) in aortic, femoral and mesenteric arterial rings which was attributed to reduced calcium activation by myofilaments^16^. In addition, resistant arteries from gluteal biopsies of CADASIL patients also showed reduced sensitivity to NA^52^ probably due to over-activity of sympathetic outflow which was also reported in sepsis^8^. In the present study, besides significant reduction in the level of NICD in septic mouse aorta, a significant down-expression in aortic Notch3 receptor was also observed in sepsis. Thus it may not be unreasonable to infer that the changes observed in the Notch targets following sepsis were attributed to the change in the activity of both Notch1 and Notch3 signalling. However, further studies are required to evaluate the changes in the NICD3 level and its downstream regulatory genes.

Gamma (γ)-secretase inhibition following DAPT treatment in mice was reported to reduce CLP-induced lethality due to reduction in inflammatory reaction in macrophages^21^. Additionally, LPS-induced inflammation and mortality were found to be lower in RBPJ^−/−^ mice as compared to their wild type litter mates due to reduction in pro-inflammatory cytokine expression^23^. To evaluate whether this beneficial effects of DAPT as reported in macrophages can also improve the vascular hyporeactivity in sepsis, we aimed to study the vascular response to NA (a commonly used vasoconstrictor to overcome refractory hypotension in sepsis) following DAPT treatment in sepsis. Although DAPT treatment did not produce significant alteration in the aortic expression of Notch3 receptor and Jag1 ligand in the present study, however, the effector genes of Notch signalling (Hey1 and MLCK) were significantly down-regulated in the aorta of SO mice following DAPT treatment. Similarly, there were marked reduction in the mRNA expressions of Hey1 (39.47%) and MLCK (89.89%) in the aorta of CLP mice following DAPT treatment as compared to that observed in the untreated septic mice. These findings evidently suggest that DAPT (γ-secretase inhibitor) treatment in the present study markedly suppressed the aortic notch signalling. Surprisingly, we did not find any improvement in the sepsis-induced vascular hyporeactivity to NA and receptor-independent contractile agents (CaCl_2_ or High K^+^) following DAPT treatment, albeit, none of these DAPT-treated septic animals were found to be dead or in morbid condition up to 20 h post-CLP. Similar to our observations, although systemic administration of γ-secretase inhibitor exhibited beneficial effect in atherosclerosis due to reduction in Notch activity in macrophages^53^, but inhibition of vascular Notch1 in absence of external stimuli was reported to aggravate leukocyte binding and over expression of pro-atherogenic molecules suggesting that reduction in the endothelial Notch1 is the predisposing factor for onset of vascular inflammation and initiation of atherosclerosis^22^. In addition, protective role of Notch signalling has been reported in several vascular injuries and diseases^54,55^. Moreover, administration of microRNA-126 in mice was found to alleviate atherosclerosis by suppressing Notch antagonist Dlk1^56^. Thus it may not be unreasonable to infer that Notch signalling plays different roles in macrophage and vascular beds in the context of inflammation and injury; thus therapies targeting systemic inhibition of Notch signalling may have serious impact especially on vascular function where basal Notch signalling plays a significant role in maintaining the physiological activity. Alternatively, pharmacological or genetic manipulation of vascular Notch signalling may be a useful novel strategy to improve the vascular reactivity in sepsis.

Overproduction of iNOS-derived NO by bacterial endotoxins and cytokines causes hyporeactivity to vasopressors resulting in cardiovascular failure in septic shock^7,8^. Further, LPS is reported to cause down-regulation of Notch signalling via iNOS/NO-mediated nitration of tyrosine residues of Notch1 protein in rat alveolar macrophages^29^. Owing to this possible cross-link between iNOS and Notch signalling, we aimed to evaluate the role of iNOS-derived NO in sepsis-induced fall in vascular Notch signalling and its targeted contractile genes. Treatment of septic mice with 1400 W (@1 mg/kg. b.wt) was reported to prevent CLP-induced cardiac hypo-responsiveness^57^. Earlier we have also reported a partial reversal of vascular hyporeactivity to NA in septic mouse aorta following in vivo treatment with 1400 W at the same dose rate^8^. In the present study, we have also observed that when septic mice were treated with 1400 W at the same dose rate as reported earlier^57^ there was significant restoration of aortic Notch3 mRNA expression suggesting a predominant role of iNOS-derived NO in sepsis-induced down-regulation of Notch3 in mouse aorta. However, we did not observe any significant change in Notch ligand (Jag1) and downstream effector (MLCK) expression following 1400 W treatment in septic mice. Unlike septic mice, inhibition of iNOS in the SO mice did not produce any significant change in the expression profiles of Notch3 receptor, Jag1 ligand and its downstream effector (MLCK) gene in the mouse aorta. Thus, further studies are warranted to unravel the mechanistic pathway responsible for down-regulation of Notch signalling and associated contractile genes in aorta of the septic mice.

## Conclusion

In conclusion, sepsis caused down-regulation of Notch signalling along with its effector genes and contractile signalling mechanism(s) in the mouse aorta. The iNOS/NO pathway is involved, at least in part, in sepsis-induced down-regulation of Notch3 receptor, however, its role in regulating the downstream effector genes needs further investigation. Further, unlike that observed in the inflammatory cells (macrophages), systemic inhibition of Notch signalling (by DAPT) did not produce beneficial effect on sepsis-induced vascular hyporeactivity. However, sepsis-mediated down-regulation of Notch3 and its contribution to vascular hyporeactivity needs to be established in future using genetic approach.

## Supplementary Information

Supplementary Information.
