# Supplementary material for: Sepsis downregulates aortic Notch signaling to produce vascular hyporeactivity in mice
Source: Sci Rep. 2022 Feb 21;12:2941. doi: 10.1038/s41598-022-06949-3 (PMC8861011; doi:10.1038/s41598-022-06949-3)
Supplement: Supplementary file 1 — Supplementary Information. [file 41598_2022_6949_MOESM1_ESM.pdf]

SUPPLEMENTARY FILE

Sepsis downregulates aortic Notch signaling to produce vascular hyporeactivity in mice

Vandana Singh[1], Raut Akash[1], Gaurav Chaudhary[1], Rajneesh Singh[1], Soumen Choudhury[1]*, Amit Shukla[1], Shyama N Prabhu[2], Neeraj Gangwar[2] and Satish K Garg[1]

[1]Smooth Muscle Pharmacology & Molecular Pharmacology Laboratory, Department of Veterinary Pharmacology & Toxicology, College of Veterinary Science and Animal Husbandry, U.P. Pandit Deen Dayal Upadhyaya Pashu Chikitsa Vigyan Vishwavidyalaya Evam Go-Anusandhan Sansthan, Mathura-281001

[2]Department of Veterinary Pathology, College of Veterinary Science and Animal Husbandry, U.P. Pandit Deen Dayal Upadhyaya Pashu Chikitsa Vigyan Vishwavidyalaya Evam Go-Anusandhan Sansthan, Mathura-281001

***Corresponding Author:**

Dr Soumen Choudhury

Assistant Professor, Department of Veterinary Pharmacology & Toxicology

College of Veterinary Science and Animal Husbandry

U.P. Pandit Deen Dayal Upadhyaya Pashu Chikitsa Vigyan Vishwavidyalaya Evam

Go-Anusandhan Sansthan (DUVASU), Mathura-281001

Mob.No: +91-9411065114

Mail id: chsoumenpharma@gmail.com

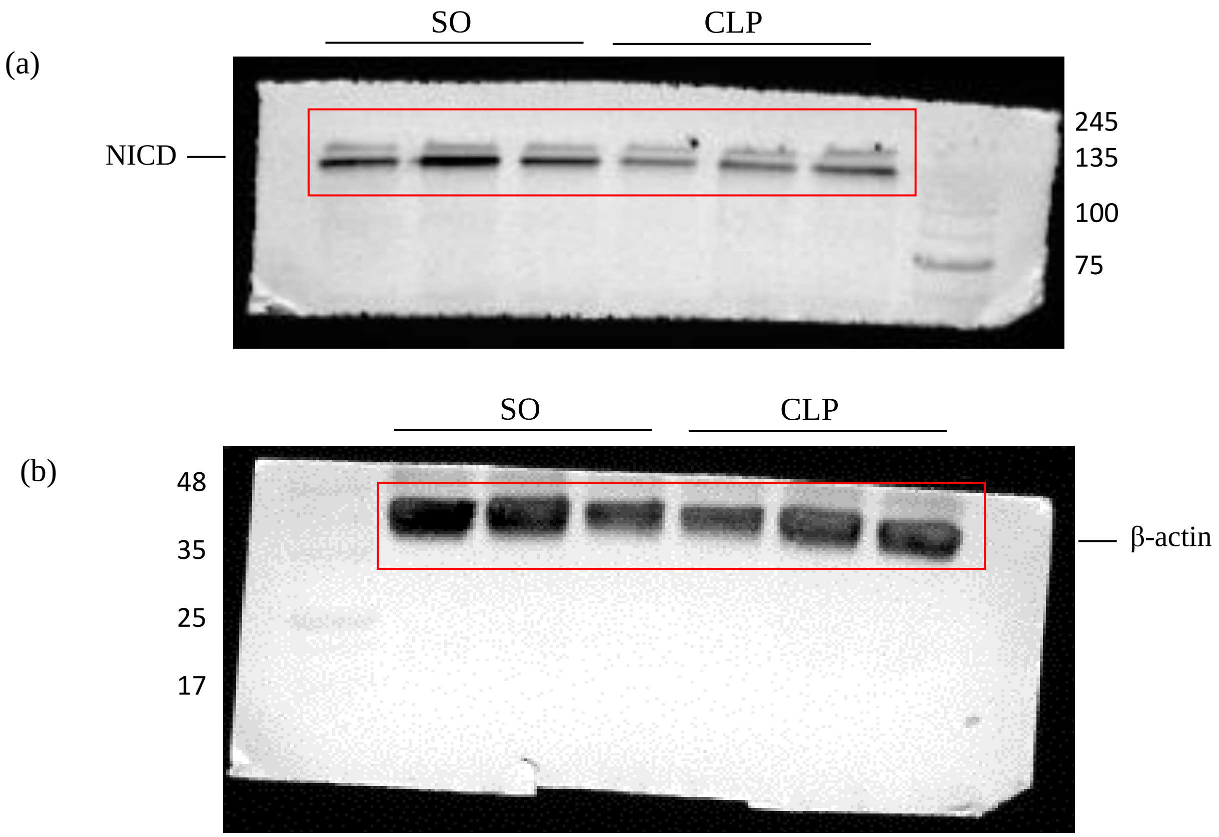

Supplementary Fig. S1: Original Western Blots.

Original blot showing the bands that corresponds to the level/expression of NICD (a) and β–actin (b). Each lane represents the individual samples.
